# Three-dimensional pelvic ultrasound is a practical tool for the assessment of anal fistula

**DOI:** 10.1186/s12876-023-02715-5

**Published:** 2023-04-25

**Authors:** Junyi Ren, Wenkun Bai, Liangrui Gu, Xiao Li, Xue Peng, WeiMei Li

**Affiliations:** 1 Department of Ultrasound in Medicine, Shanghai Jiao Tong University Affiliated 6Th People’s Hospital, Shanghai Institute of Ultrasound in Medicine, Shanghai, China; 2 Department of Ultrasound in Medicine, Shanghai Jiao Tong University Affiliated 6Th People’s Hospital, Shanghai, China; 3 Department of Radiology in Medicine, Shanghai Jiao Tong University Affiliated 6Th People’s Hospital, Shanghai, China; 4 Department of Breast Surgery, Shanghai Jiao Tong University Affiliated 6Th People’s Hospital, Shanghai, China

**Keywords:** Anal fistula, Pelvic floor ultrasound, Three-dimensional ultrasound, MRI

## Abstract

**Objective:**

This study aims to investigate the diagnostic value of three-dimensional pelvic ultrasound in the preoperative assessment of anal fistula compared with findings of MRI and surgery.

**Methods:**

A total of 67 patients (62 males) with suspected anal fistula were analyzed retrospectively. Preoperative three-dimensional pelvic ultrasound and magnetic resonance imaging were performed in all patients. The number of internal openings and the type of fistula were recorded. The accuracy of three-dimensional pelvic ultrasound was determined by comparing these parameters with surgical outcomes.

**Results:**

At surgery, 5 (6%) were extrasphincteric, 10 (12%) were suprasphincteric, 11 (14%) were intersphincteric, and 55 (68%) were transsphincteric. There was no significant difference in the accuracy of pelvic 3D US and MRI, based on internal openings (97.92%, 94.79%), anal fistulas (97.01%, 94.03%), and those under Parks classification (97.53%, 93.83%).

**Conclusion:**

Three-dimensional pelvic ultrasound is a reproducible and accurate method for determining the type of fistula and detecting internal openings and anal fistulas.

## Introduction

Anal fistula is a type of common perianal inflammatory disease that is second only to hemorrhoids in prevalence and accounts for 3% of all anorectal diseases [1]. It is an abnormal hollow tract connecting an internal opening in the anal canal to an external opening in the perianal skin [2], causing pain, drainage of pus or stool, pruritus, excoriation of adjacent tissue and other symptoms, all of which negatively affect the patient’s quality of life [3].

The main treatment method for anal fistula is surgical therapy [4], which has the potential to eradicate anal fistula while preserving anal continence. After surgery, however, the recurrence rate of anal fistula is 0–26.5%, which could be due to the failure to accurately identify the internal opening, primary tract and fistula branch before surgery [5]. The accurate detection and classification of these lesions are critical for effective treatment and reducing recurrence rates. As a result, precise imaging examination is of great significance. Current diagnostic methods include anorectal ultrasound (AUS), computed tomography (CT), endoscopic ultrasound (EUS), and magnetic resonance imaging (MRI) [6].

MRI has been identified as the preferred diagnostic method for detecting anal fistula with high specificity and sensitivity. Pelvic ultrasonography has been proposed as a low-cost, easily accessible, noninvasive and accurate tool for dynamic and static assessment of anal canal anatomy and function. Multislice imaging is now possible with the introduction of three-dimensional ultrasound [7], allowing for a comprehensive assessment of sphincter and anal canal. Therefore, this study aims to detect the use of pelvic 3D US in determining anal fistula and measure the diagnostic value of US in locating internal openings and classifying anal fistulas.

## Methods

### Subjects

This is a retrospective analysis of 67 patients with suspected anal fistula who underwent surgery between May 2021 and July 2022. The enrolled patients included 62 men and 5 women with a mean age of 35.40 ± 13.29 (16–68). The scheme was conformed to the Declaration of Helsinki and approved by the Ethics Committee of Shanghai Jiaotong University. Patients who defaulted to preoperative pelvic 3D US and MRI were excluded from the study.

### Exclusion criteria

Exclusion criteria were: (1) patients who underwent seton treatment or surgery before pelvic 3D US and MRI; (2) patients with serious or uncontrolled infection, low endurance for US and MRI, and severe cardiopulmonary dysfunction and disorder.

### Instruments

#### Ultrasonographic assessment

The pelvic 3D US technique (WS80A; Samsung Medical) was used to obtain high-resolution images of the complex anatomy of sphincter and anal canal. A rotating probe (CA1-8A; Samsung Medical) was used to obtain 360° US images of the area of interest from patients in a left lateral position and lithotomy position.

### MRI assessment

Patients were examined in a eupneic supine position. T1-weighted (T1W1) and T2-weighted (T2W2) sequences on a 3.0 Tesla scanner (Philips Ingenia) were used to obtain images of three orthogonal imaging planes (sagittal, coronal and axial).

The position and number of internal openings, major fistula trend and Parks classification were documented in the above examination. The pelvic 3D US and MRI were analyzed and evaluated by two experienced doctors with more than 10 years of experience.

### Assessment criteria

#### Diagnosis of anal fistula using pelvic 3D US

An anal fistula is a low echo duct that connects the anal canal or rectum to the perianal skin, with one or more internal openings, sometimes accompanied by one or two external openings. When tracing an anechoic or hypoechoic zone inward, the pelvic 3D US image of a fistula shows an anechoic or hypoechoic zone with internal openings. The US image of internal openings shows local mucosal depression, local mucosal protrusion or mucous membrane disruption. The external and internal anal sphincters appear as two concentric rings, with the external anal sphincter as a hyperechoic outer layer and the internal anal sphincter as a hypoechoic inner layer.

### Diagnosis of anal fistula using MRI

The fistula and its branches are present in T1W1 as streaks with low or equal signal intensity. The fistula is on one side of the internal opening, while the rectal cavity is on the other side. MRI suggests local mucosal disruption and protrusion with high T1 and T2 signal intensity.

### Surgical treatment

Patients who had been clinically diagnosed anal fistula underwent surgery. Anal fistula resection was performed on all patients with low perianal fistulas, while thread drawing was performed on all patients with high perianal fistulas. The postoperative pathological results confirmed the type of perianal fistula, the presence and localization of the internal opening, primary tract, secondary extension, abscess, and pelvic floor muscle condition. All patients received a detailed pathology report.

### Parks classification system

Park’s classification is an anatomically precise classification developed for surgical use based on the relationship between the fistula and the external sphincter [8]. This classification divides anal fistulas into four types: inter-sphincteric (type I), trans-sphincteric (type II), supra-sphincteric (type III), and extra-sphincteric (type IV). Although less common, types III and IV have been reported as related to a higher risk of recurrence and complication [9].

### Statistical analysis

The SPSS version 26 system (IBM, The United States of America) was used for statistical analysis. Categorical variables were compared using the χ2 test, while measurement data were compared using the t-test. Categorical data were represented by numbers (%), and measurement data were represented by mean ± standard deviation. The gold standard was the surgical diagnosis. The accuracy of MRI and pelvic 3D US was calculated for the diagnosis of anal fistulas, internal openings and Parks criteria. The level of statistical significance was set at *P* < 0.05.

## Results

### The accuracy rate of MRI and pelvic 3D US in diagnosing anal fistula

**Fig. 1:**
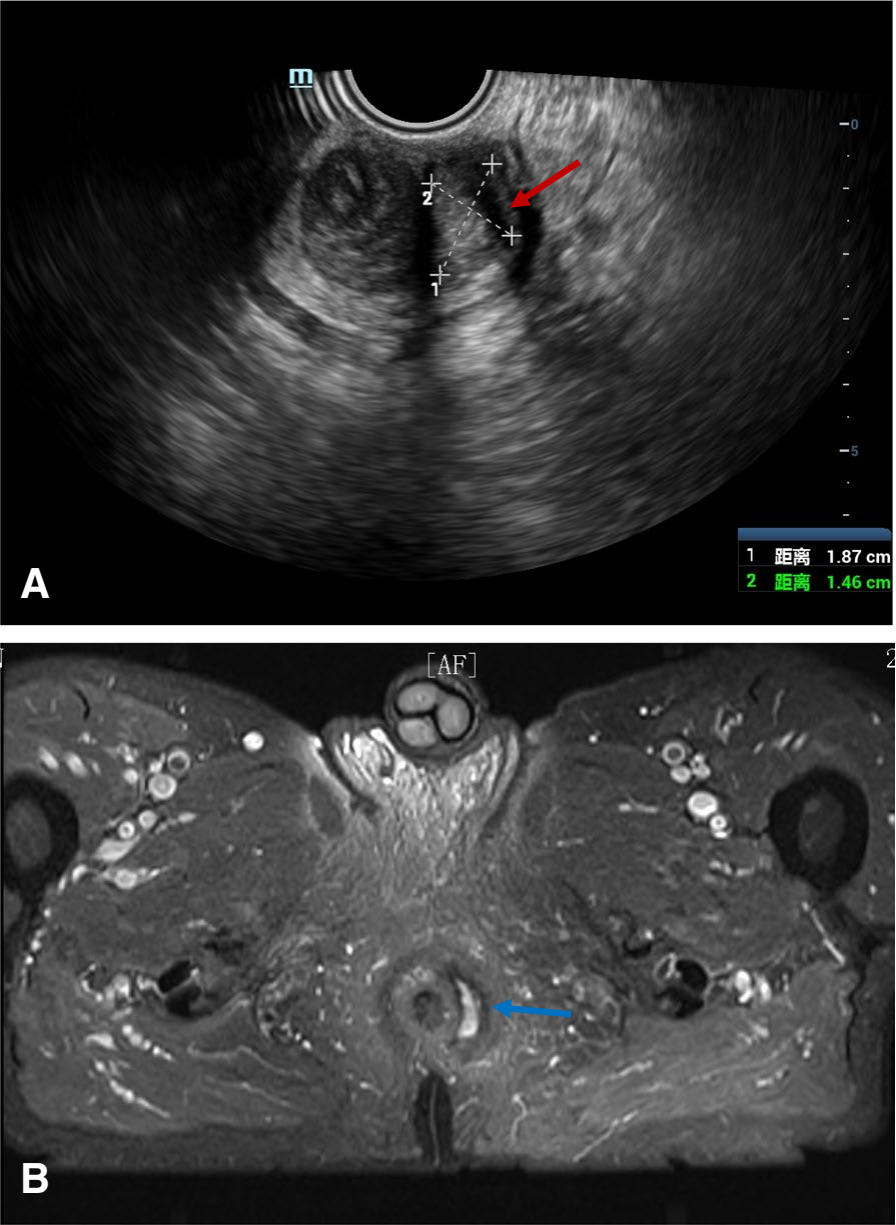
Anal fistula type I in pelvic 3D US and MRI images. **A** Pelvic 3D US image of a anal fistula(red arrow); (**B**)MRI image of a anal fistula(blue arrow)

**Fig. 2:**
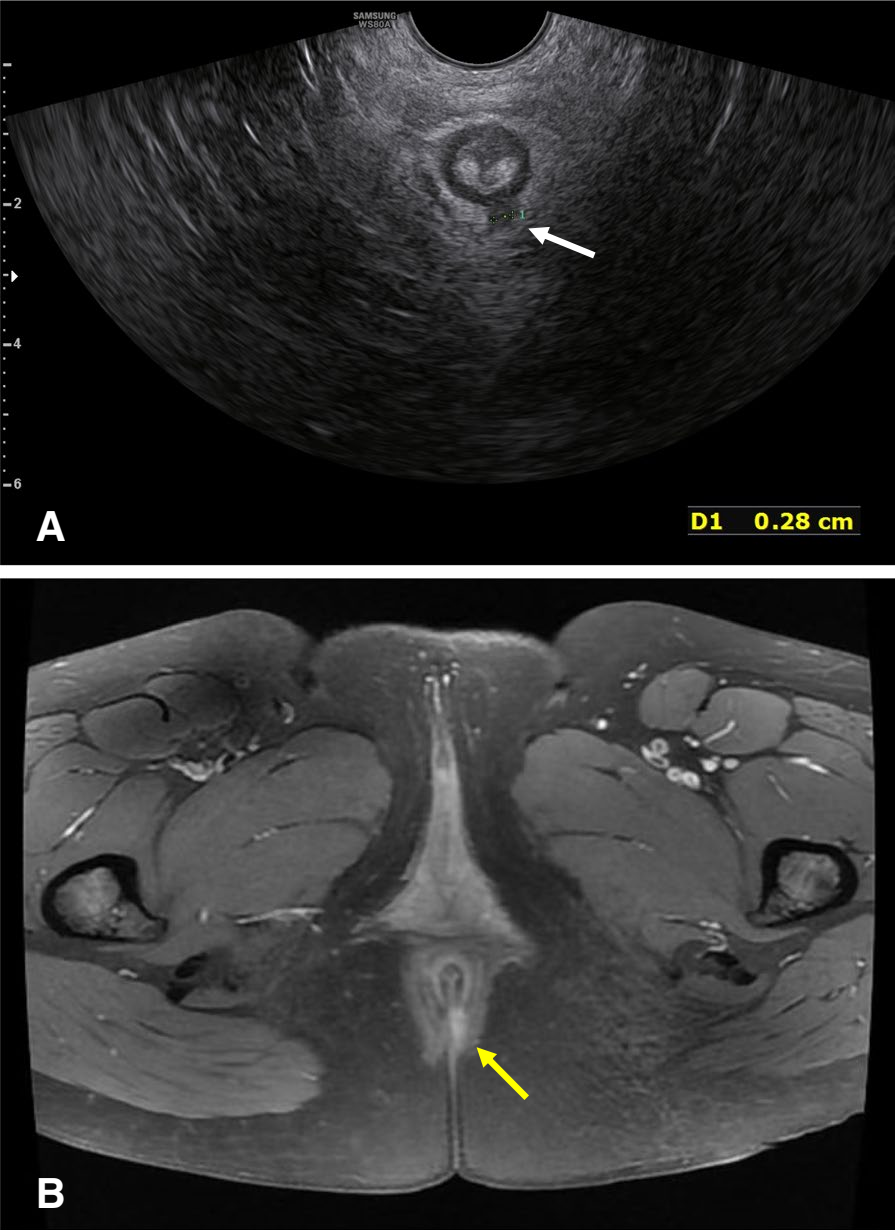
Anal fistula type II in pelvic 3D US and MRI images. **A** Pelvic 3D US image of a anal fistula(white arrow); (**B**)MRI image of a anal fistula(yellow arrow)

**Fig. 3:**
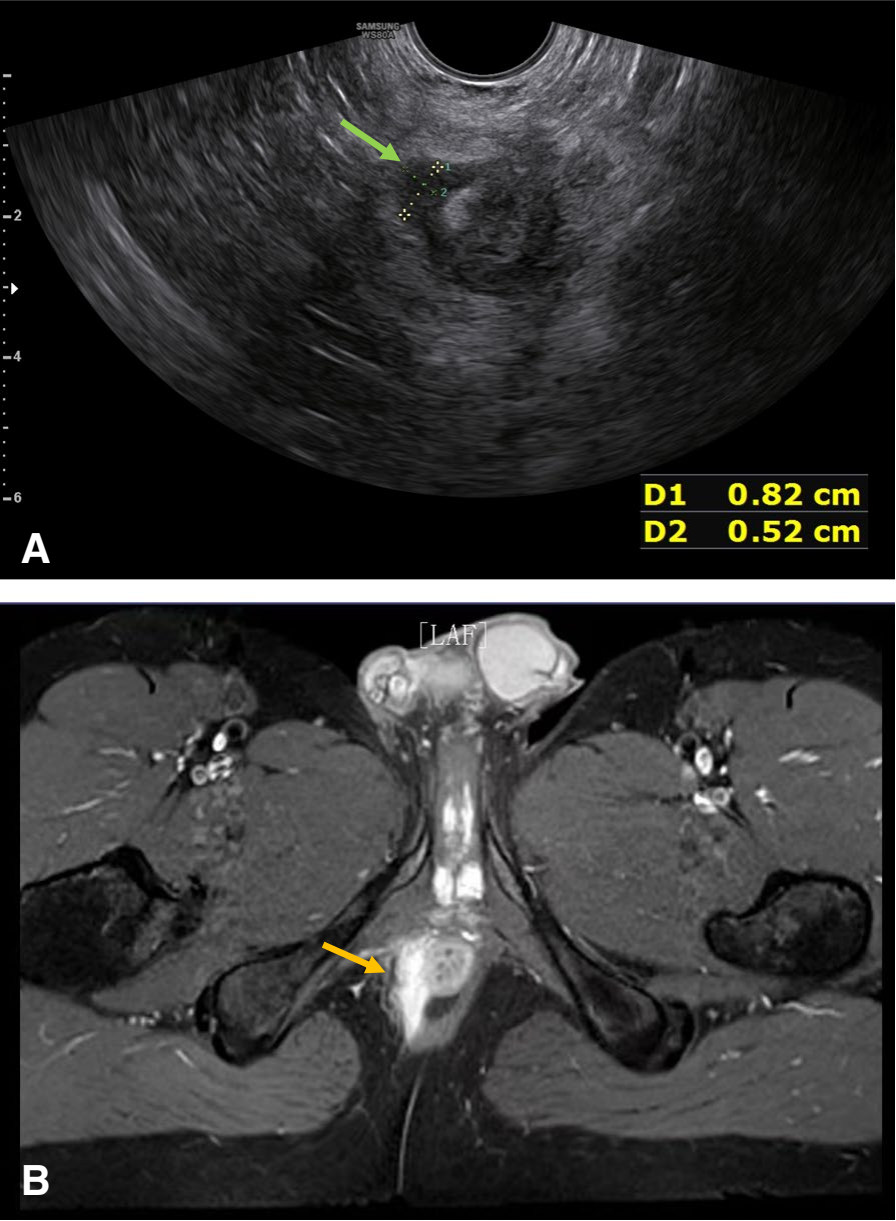
Anal fistula type III in pelvic 3D US and MRI images. **A** Pelvic 3D US image of a anal fistula(green arrow); (**B**)MRI image of a anal fistula(orange arrow)

**Fig. 4:**
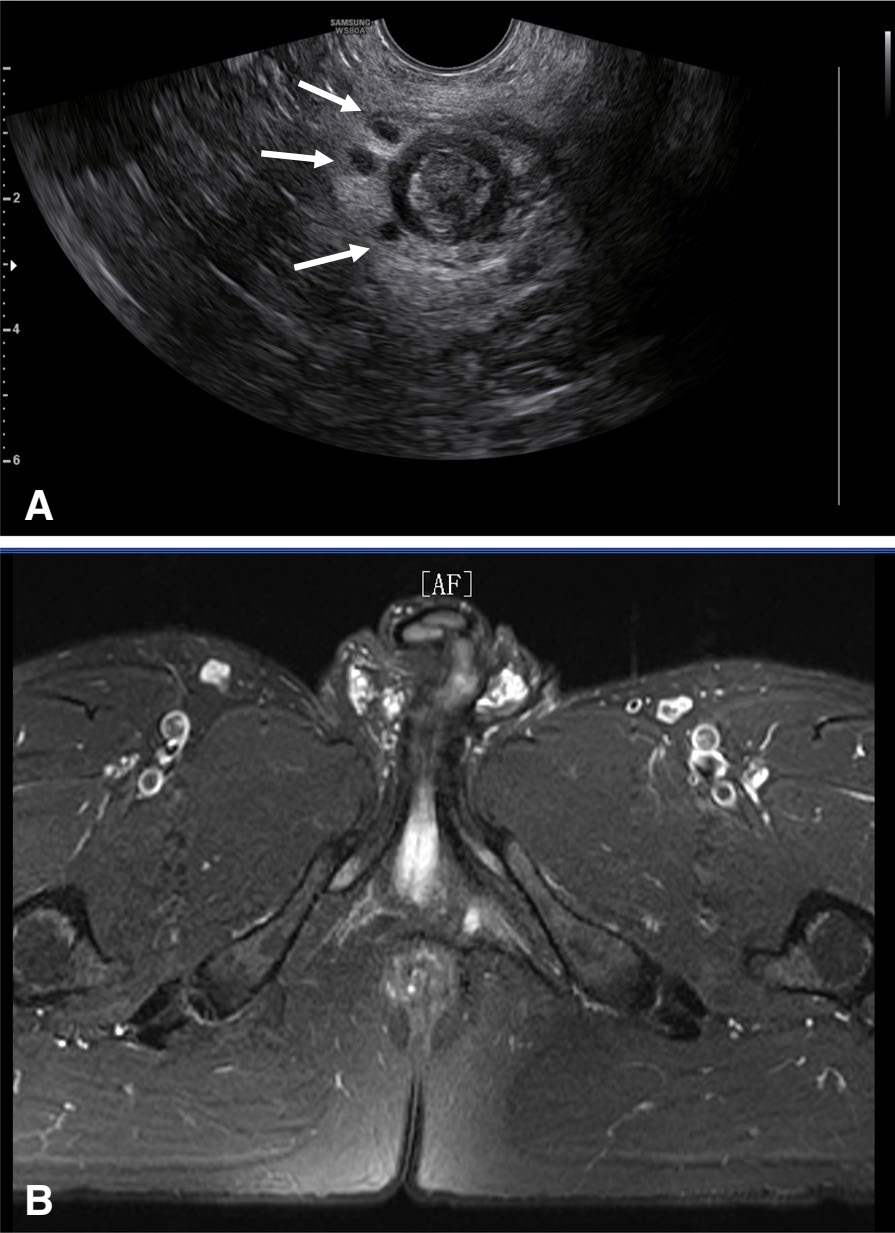
Anal fistula type II and IV in pelvic 3D US and MRI images. **A** Pelvic 3D US image of internal openings(white arrow); (**B**)MRI image of a anal fistula

In this study, the anal fistula of the same patient was measured using pelvic 3D US and MRI (Figs. 1, 2, 3 and 4). The reference standard was surgical treatment. The number of correct MRI/US diagnoses or the total number of lesions was used to calculate accuracy.

Among 67 surgical patients, 65 anal fistulas were discovered intraoperatively, 63 were detected by MRI, and 65 were detected by pelvic 3D US. Based on the surgical findings, there was no statistically significant difference in the accuracy of pelvic 3D US and MRI in identifying anal fistula correctly.

The overall accuracy of MRI and pelvic 3D US in detecting anal fistula is shown in Table 1.

**Table 1:** The accuracy rates (%) in diagnosing anal fistula by MRI and pelvic 3D US

Variables	MRI(%)		Pelvic 3D US(%)	*P* value
accuracy rate(n)	63/67(94.03)		65/67(97.01)	0.625

### The accuracy rate of MRI and pelvic 3D US in classifying anal fistula

Of 67 surgical patients, 7 were classified as Parks I, 55 as Parks II, 10 as Parks III, and 5 as Parks IV. The accuracy rates of MRI and pelvic 3D US were 93.83% (76/81) and 97.53% (79/81), respectively, with no statistically significant difference (*P* = 0.375, Tables 2 and 3).

**Table 2:** MRI-base and pelvic 3D US -base parks classification

MRI scan					Pelvic 3D US			
Is*	Ts*	Ss*	Es*		Is*	Ts*	Ss*	Es*
10	0	0	0		11	0	0	0
0	51	0	0		0	53	0	0
0	0	10	0		0	0	10	0
0	0	0	5		0	0	0	5

Is* Intersphincteric fistula, Ts* Transsphincteric fistula, Ss* Suprasphincteric fistula, Es* Extrasphincteric fistula

**Table 3:** The accuracy rates (%) in classifying anal fistula by MRI and pelvic 3D US

Variables	MRI(%)		Pelvic 3D US(%)	*P* value
accuracy rate(n)	76/81(93.83)		79/81(97.53)	0.375

Table 2 describes the findings of MRI and pelvic 3D US concerning the classification of anal fistula. The overall accuracy of MRI and pelvic 3D US in classifying anal fistula is shown in Table 3.

### The accuracy rate of MRI and pelvic 3D US in detecting internal openings

Of 67 surgical patients, 46 had one internal opening each, 16 had two internal openings each, 3 had three internal openings each, 1 had four internal openings, and 1 had five internal openings. The accuracy rates of MRI and pelvic 3D US were 94.79% (91/96) and 97.92% (94/96), respectively, with no statistically significant difference (*P* = 0.375, Tables 4 and 5).

**Table 4:** MRI-base and pelvic 3D US -base internal openings

Internal opening(n)	Surgery(n)	MRI(%)	Pelvic 3D US(%)
1	46	43/46(93.48)	44/46(95.65)
2	16	15/16(93.75)	16/16(100.00)
3	3	3/3(100.00)	3/3(100.00)
4	1	1/1(100.00)	1/1(100.00)
5	1	1/1(100.00)	1/1(100.00)

**Table 5:** The accuracy rates (%) in diagnosing internal opening by MRI and pelvic 3D US

Variables	MRI(%)		Pelvic 3D US(%)	*P* value
accuracy rate(n)	91/96(94.79)		94/96(97.92)	0.375

Table 4 describes the findings of MRI and pelvic 3D US concerning the identification of internal openings. The overall accuracy of MRI and pelvic 3D US in detecting internal openings is shown in Table 5.

## Discussion

Surgery is the most basic treatment option for anal fistula. However, the high recurrence rate of anal fistula is a common problem, especially in recurrent and complex fistulas. For effective surgical management, prior knowledge of the fistula site, the main type of perianal fistula, and the anatomy of internal openings before surgery is critical to improving the success rate of one-time surgery. Some of the most popular imaging assessment modalities include fistulography, endoscopic ultrasound, CT, MRI, and B-ultrasound.

Fistulography is limited due to its low diagnostic accuracy [10], which is unable to show the anal sphincter or establish its relationship with the fistula [11]. The extension away from the primary track may also be difficult. Endosonography allows for detailed anal anatomy visualization with high spatial resolution and can be used to classify the type of fistula [12]. However, suprasphincteric or secondary tracts cannot be detected due to the endosonography’s limited field of view [11]. Conventional 2D CT image is still insufficient for a detailed analysis of fistulous anatomy and accurate classification. It also cannot fully depict the distribution of subtle fistula due to its poor capacity of soft tissue differentiation [13, 14]. In addition, CT subjects patients to ionizing radiation [15].

MRI is a highly accurate noninvasive modality for detecting and characterizing the presence and location of the primary fistulous track, secondary extension and accompanying abscess, as well as delineating its extent [16, 17]. Due to its high spatial resolution and soft tissue contrast in the perianal region, MRI has become the preferred imaging modality for assessing perianal fistula [18]. Preoperative MRI has been shown to influence subsequent surgery and, as a result, significantly reduce the risk of recurrence [12]. However, it remains constrained by cost and accessibility [19]. It is also time-consuming to obtain multiple sequences for depicting the fistula in detail [6]. The activity of the fistula or abscess is also thought to play a role in determining the surgical treatment strategy.

Recently, DW-MRI has been recommended to aid in diagnosis because it can detect the presence and extent of a fistula. Furthermore, it is a useful tool for assessing the activity of the anal fistula. However, due to the low spatial resolution, it is unable to evaluate the course of the fistulous track concerning adjacent structures [20].

Ultrasound examination of the anal fistula is a real-time, high-resolution, effective and safe method for evaluation, which has been developed as an alternative imaging technique. Unlike MRI, ultrasound is well tolerated by patients and provides dynamic anatomy and orientation of the fistula tract. In addition, it can be also intraoperatively used to aid in surgical management. Due to the limitation of viewing images in only one plane, three-dimensional ultrasound has been recently introduced for high-resolution imaging of the anal canal and anal sphincter anatomy on multiple planes. The number and location of fistulas and internal openings, as well as a focal defect in the anal canal mucus and its communication with a large superficial abscess, can be precisely delineated. Three-dimensional anorectal ultrasound has improved the diagnosis of anal fistula by providing a detailed multiplanar reconstruction of the anal canal, with higher accuracy in demonstrating the relationship between anal sphincter and fistula tract. It also increases the detection sensitivity of fistula tracks, internal openings and anal sphincter defects, which is important in surgical planning to minimize damage to the anal sphincter complex. However, the pain of patients can be exacerbated when they are evaluated with a three-dimensional intracavitary probe. Thus, three-dimensional pelvic ultrasound can be used to alleviate pain and operate around the subcutaneous abscess without the need for anal expansion.

This study demonstrates that using three-dimensional pelvic ultrasound to assess anal fistula can provide a multiplane preoperative mapping of perianal fistula, identify all components (such as the position and type of primary and secondary tracts, and internal openings), quantify the length of the injured sphincter, display the relationship between sphincter and fistula, and classify anal fistula. This method is well-tolerated and minimally invasive. The results indicated the accuracy rates of pelvic 3D US and MRI for assessing internal openings (97.92%, 94.79%), perianal fistulas (97.01%, 94.03%), and those under Parks classification (97.53%, 93.83%), respectively, with no statistically significant difference (*P* > 0.05). This suggests that the two diagnostic methods are equally effective in the diagnosis of anal fistula.

This study has a small sample size. Due to transonic beam penetration restriction and potential air interference, ultrasound has limitations in detecting and imaging deep lesions [21]. Supralevator fistula is the most complex and rarest type of anal fistula. A larger sample size could be used to assess the availability of pelvic 3D US imaging in the diagnosis of high anal fistula. Furthermore, the follow-up period is required to provide a reasonable assessment of fistula healing rates.

## Conclusion

In conclusion, three-dimensional pelvic ultrasound demonstrates high accuracy in the diagnosis of fistula positioning, internal opening and Parks classification, allowing for a safe treatment approach.

## Data Availability

The datasets generated during the current study are available from the corresponding author on reasonable request.
